# The Brain–Gut Health Initiative (BIGHI): A Prospective Cohort on Psychiatric Disorders in China

**DOI:** 10.34133/research.1142

**Published:** 2026-03-03

**Authors:** Fengchun Wu, Baoyuan Zhu, Shixuan Feng, Hehua Li, Jing Zhou, Yuping Ning, Yuanyuan Huang, Kai Wu

**Affiliations:** ^1^ Department of Psychiatry, The Affiliated Brain Hospital, Guangzhou Medical University, Guangzhou 510370, China.; ^2^ School of Biomedical Sciences and Engineering, South China University of Technology, Guangzhou International Campus, Guangzhou 511442, China.; ^3^ School of Materials Science and Engineering, South China University of Technology, Guangzhou 510006, China.; ^4^ Guangdong Engineering Technology Research Center for Translational Medicine of Mental Disorders, Guangzhou 510370, China.; ^5^ National Engineering Research Center for Tissue Restoration and Reconstruction, South China University of Technology, Guangzhou, 510006, China.; ^6^ Institute of Gerontology, Guangzhou Geriatric Hospital, Guangzhou Medical University, Guangzhou, China.; ^7^ Collaborative Innovation Center for Civil Affairs of Guangzhou, Guangzhou, China.; ^8^ Department of Aging Research and Geriatric Medicine, Institute of Development, Aging and Cancer, Tohoku University, Sendai 980-8575, Japan.

## Abstract

Major psychiatric disorders are characterized by substantial clinical heterogeneity and high comorbidity, yet their underlying biological mechanisms are not fully uncovered. The microbiota–gut–brain axis (MGBA) offers a cross-system perspective for elucidating the pathophysiology of major psychiatric disorders. The Brain–Gut Health Initiative (BIGHI) was established as the first prospective longitudinal cohort in China dedicated to investigating major psychiatric disorders guided by the framework of MGBA, enabling large-scale, transdiagnostic, and longitudinal analyses of brain–gut interactions. To date, the BIGHI has enrolled over 1,200 participants with schizophrenia, major depressive disorder, bipolar disorder, and healthy controls, with multidimensional data collected including clinical symptomatology, neurocognitive performance, electroencephalography, magnetic resonance imaging, peripheral blood biomarkers, and gut microbiome profiles. The studies within the BIGHI reveal (a) brain–gut physiological alterations in psychiatric disorders; (b) systematic relationships among brain function, peripheral physiological markers, and gut microbiome; and (c) brain–gut network patterns with marked interindividual heterogeneity. In future studies, we will expand the BIGHI into a collaborative network and promote data harmonization and interdisciplinary collaboration to advance computational psychiatry as well as its clinical translation.

## Introduction

Psychiatric disorders represent a growing global health burden, affecting nearly 10% of the population worldwide and substantially impacting approximately one-quarter of families [1]. Over the past 30 years, China has experienced unprecedented economic development and social transformation. These transformations have driven major shifts in demographics, urbanization, mobility, education, and disease epidemiology, with significant implications for the onset and management of psychiatric disorders [2]. According to a nationally representative epidemiological survey of psychiatric disorders in China, the lifetime prevalence of any psychiatric disorder (excluding late-life dementia) among adults is 16.57%, with anxiety and mood disorders being the most common. Notably, severe psychiatric disorders such as schizophrenia (SZ), major depressive disorder (MDD), and bipolar disorder (BD) impose a disproportionate public health burden due to their chronic course, high disability rates, and substantial treatment gaps [3]. Furthermore, according to the latest report from the World Health Organization, the global burden of psychiatric disorder has significantly increased following the COVID-19 pandemic. During the COVID-19 pandemic, the number of individuals with SZ, MDD, and other psychiatric disorder has increased by 25% to 27.6%, and the pandemic’s impact on mental health is expected to last for at least 2 decades [4]. As this long-term impact continues, the prevalence of psychiatric disorders in China continues to rise, but the field of mental health still faces multiple challenges, such as insufficient public awareness, a lack of medical resources, and weak professional capacity. More critically, diagnosis remains primarily reliant on subjective clinical assessments, lacking objective biomarkers, which hinders accurate disease identification and timely intervention [5].

In recent years, a growing body of study has highlighted the importance of gut–brain communication in maintaining host health [6–8], and the microbiota–gut–brain axis (MGBA) offers a new perspective for understanding the origins and development of psychiatric disorders [9]. It represents a complex bidirectional communication network between the gut and the brain involving neural pathways immune signaling and microbial-derived metabolites such as neurotransmitter precursors and short-chain fatty acids that collectively regulate host health [10]. Accumulating evidence now suggests that the MGBA plays a potential role in the development and progression of psychiatric disorders [11]. The integrity and functionality of the MGBA are shaped by genetic, environmental, dietary [12], pharmacological, and psychosocial factors [13], highlighting the need to elucidate its mechanisms to better understand and treat psychiatric disorders [14,15].

To better understand how these factors influence psychiatric disorders, numerous studies have investigated gut microbiome profiles in relation to brain imaging, revealing disease-associated alterations in the MGBA. For instance, alterations in the gut microbiome of patients with SZ, including changes in the family *Bifidobacteriaceae*, were observed and found sufficient to differentiate SZ patients from healthy controls (HC). Experimental studies further support a causal role for these microbial changes, as fecal microbiota from patients with SZ could induce behavioral abnormalities such as hyperactivity and anxiety in mice [16]. Similarly, a clinical study involving 97 individuals reported significant differences in the composition and diversity of the gut microbiome between BD patients and HC [17]. After the treatment with quetiapine, significant changes in the *Bacteroidetes* phylum of patients with BD were observed. Subsequent receiver operating characteristic (ROC) analysis demonstrated that the abundance of *Bacteroidetes* could effectively differentiate treatment responders from nonresponders. Additionally, fecal microbiota from rats exhibiting negative behaviors were shown to induce depression-like symptoms in recipient mice, indicating a key role of the gut microbiome in stress vulnerability [18].

To overcome the limitations of single-modality approaches, studies have increasingly combined gut microbiome profiling with brain imaging, providing a more comprehensive understanding of MGBA function. A recent genome-wide association analysis of 5 psychiatric disorders, including SZ and BD, reported that measures of brain white matter integrity mediated the association between variation in the gut microbiome and psychiatric conditions [19]. Similarly, studies on healthy individuals have shown that gut microbiome is associated with brain structure and function [20]. These studies highlight the pivotal role and potential application of the MGBA hypothesis in understanding the pathophysiological mechanisms of various psychiatric disorders and in the development of diagnostic and therapeutic approaches.

Despite growing evidence linking the MGBA to psychiatric disorders, most clinical studies are cross-sectional, limiting causal inference between gut dysbiosis and brain dysfunction, and failing to capture dynamic changes across disease progression, thus hindering clinical translation [21]. Additionally, several population cohorts centered on gut microbiota have been preliminarily established both domestically and internationally [22], alongside some large-scale cohorts focused on functional magnetic resonance imaging (MRI), laying the groundwork for research in this field [23]. Additionally, the China Brain Multi-omics Atlas Project has been proposed as a large-scale research initiative aimed at constructing a human brain molecular reference atlas for East Asian populations through the integration of multi-omics data [24]. However, existing research predominantly relies on single-modality or limited omics approaches, with most cohorts constructed independently around isolated aspects of the gut microbiome, neuroimaging, or clinical phenotypes. Such designs lack systematic integration of multi-system biological information within the same individuals and a unified temporal framework. In the context of psychiatric disorders, which are highly heterogeneous and dynamically evolving, single-modality data are often insufficient to capture the holistic biological architecture of patients or to characterize dynamic changes associated with disease progression and therapeutic interventions.

**Fig. 1.**
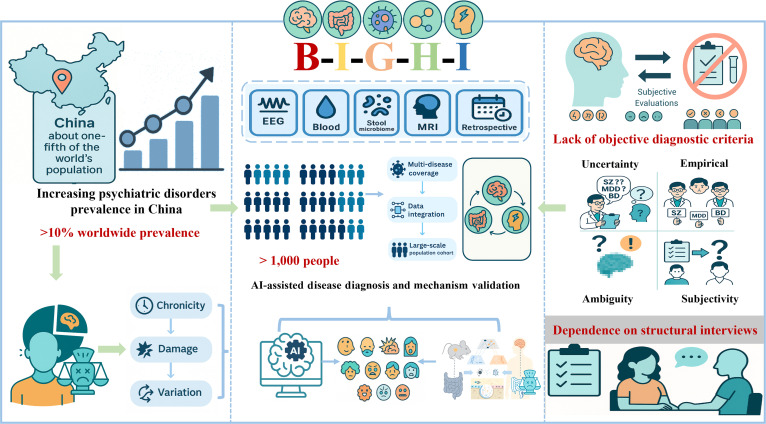
Overview of the BIGHI cohort. The figure illustrates the rising burden of psychiatric disorders in China and the limitations of current diagnoses relying on subjective interviews. BIGHI integrates multidimensional data, including MRI, EEG, blood biomarkers, and gut microbiota—from over 1,000 participants to support mechanistic studies and AI-assisted diagnosis.

To overcome this critical bottleneck, we established the Brain–Gut Health Initiative (BIGHI), the first cohort in China systematically focused on the MGBA, encompassing major psychiatric disorders including SZ, MDD, and BD, as well as HC. To date, the BIGHI cohort has integrated multidimensional data from more than 1,000 individuals, encompassing standardized clinical symptom ratings, neurocognitive assessments, electroencephalography (EEG), MRI, gut microbiome profiles, and blood-based biochemical indicators, all collected within a unified clinical and temporal framework. This high-quality dataset provides a solid foundation for systematically investigating dynamic MGBA-related processes in psychiatric disorders and is grounded in a series of our prior studies. These studies, conducted during cohort development, established and validated feasible strategies for multimodal data acquisition, integration, and analysis, yielding an operational framework for understanding multisystem co-dysregulation and supporting subsequent cross-system modeling and biomarker research (Fig. 1).

## Cohort Description

The BIGHI is a longitudinal cohort study conducted in 5 distinct phases, each targeting specific psychiatric populations and employing tailored data collection strategies (Table 1). The following sections detail the study participants and corresponding data acquisition methods.

**Table 1.** BIGHI substudies and corresponding objectives and patient cohorts

Group	Measures	Sample		Data collection				
		Number	Subjects	Clinical and MCCB	Blood	MRI	EEG	Fecal
A	Longitudinal	230 (70) ^a^	FEMDD	✓	✓	✓	✓	✓
B	Longitudinal	279 (187) ^a^	MDD	✓	✓	–	✓	–
C	Cross-sectional	63	BD	✓	✓	✓	✓	–
D	Longitudinal	321 (69) ^a^	SZ	✓	✓	✓	✓	✓
E	Cross-sectional	373	HC	✓	✓	✓	✓	✓

FEMDD, first-episode major depressive disorder; MDD, major depressive disorder; BD, bipolar disorder; SZ, schizophrenia; HC, healthy controls; MCCB, Measurement and Treatment Research to Improve Cognition in SZ (MATRICS) Consensus Cognitive Battery; MRI, magnetic resonance imaging; EEG, electroencephalogram

^a^ Baseline subject number (follow-up subject number).

### Study subjects

#### Participant recruitment

**Fig. 2.**
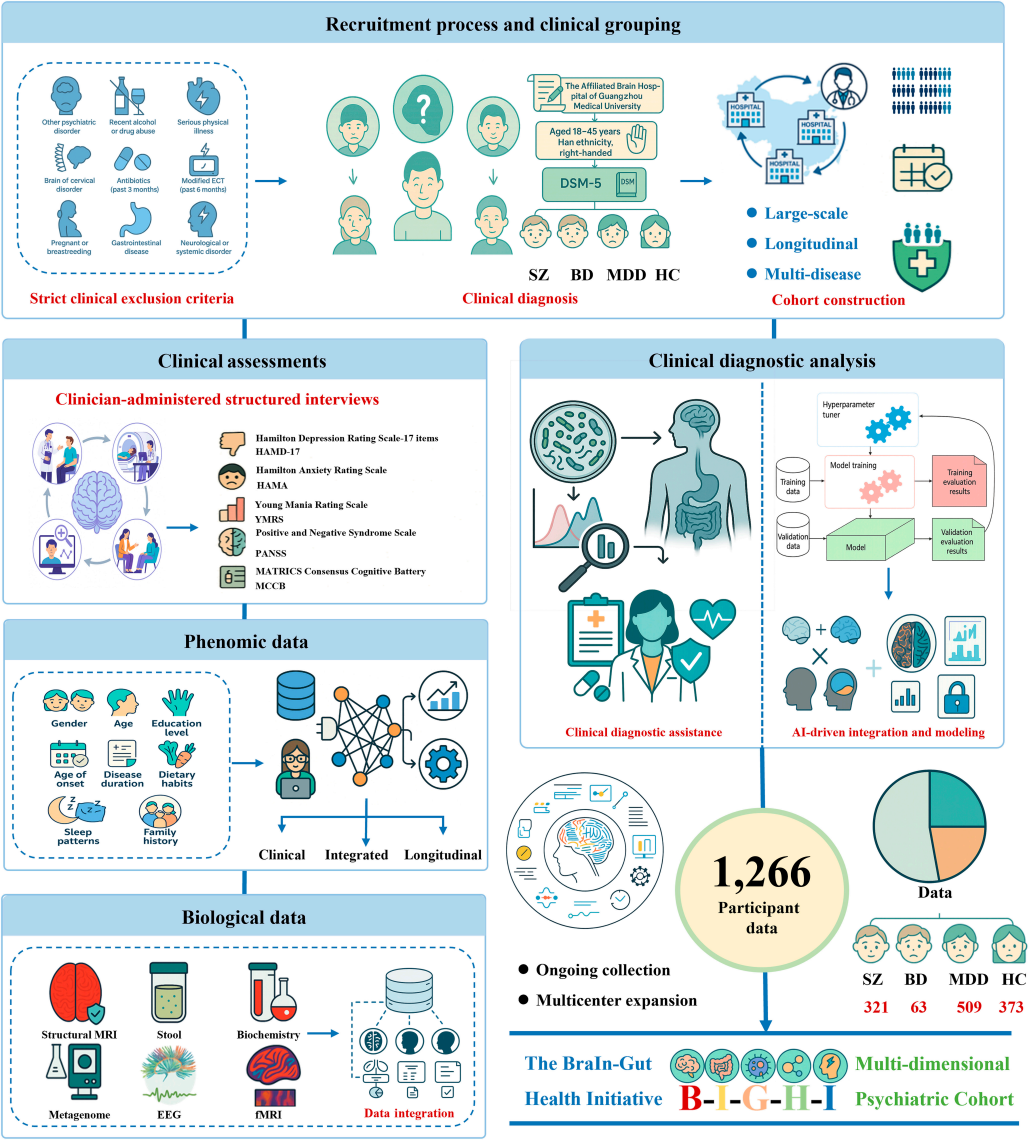
Study design framework of the BIGHI cohort. This figure illustrates the overall construction workflow and data architecture of the BIGHI multidimensional psychiatric cohort. Participants aged 18 to 45 years were recruited from the Affiliated Brain Hospital of Guangzhou Medical University and screened using strict clinical exclusion criteria. Based on *DSM-5* diagnostic guidelines, individuals were assigned to 4 groups: SZ, BD, MDD, and HC. All participants underwent clinician-administered structured interviews and completed comprehensive symptom and cognitive assessments, including the HAMD-17, HAMA, YMRS, PANSS, and MCCB. In addition, extensive phenomic information was collected, covering demographic characteristics, illness course, lifestyle factors, sleep and dietary patterns, and family history. The cohort integrates a wide range of multimodal biological data—structural and functional MRI, EEG, blood biochemical indicators, and fecal metagenomic sequencing, providing a high-dimensional foundation for investigating MGBA mechanisms from the perspectives of brain function, immune-metabolic status, and microbial ecology. Leveraging these multimodal data, the cohort supports an AI-driven analytical framework that encompasses data preprocessing, feature extraction, model training, and validation, enabling diagnostic modeling and biomarker discovery.

Participants were recruited from The Affiliated Brain Hospital of Guangzhou Medical University and included individuals diagnosed with SZ, BD, MDD, and HC. This study was approved by the Ethics Committee of the Affiliated Brain Hospital of Guangzhou Medical University and was conducted in accordance with the latest version of the Declaration of Helsinki (2013). All participants met the following general criteria: aged between 18 and 45 years, of Han ethnicity, right-handed, and provided written informed consent. Psychiatric diagnoses were determined based on the *Diagnostic and Statistical Manual of Mental Disorders, Fifth Edition* (*DSM-5*) and were validated through the Structured Clinical Interview for DSM conducted by trained clinicians. SZ patients were required to have a Positive and Negative Syndrome Scale (PANSS) total score ≥ 60, with at least 2 items scoring ≥ 4 on the positive symptom subscale. BD participants met *DSM-5* diagnostic criteria for BD and were categorized based on their current mood state at the time of assessment, including (a) manic-state BD, defined as meeting *DSM-5* criteria for a hypomanic or manic episode with a Young Mania Rating Scale (YMRS) score > 20, and being medication-naïve or free of psychotropic medications for at least 6 months; (b) depressive-state BD, defined as meeting *DSM-5* criteria for a depressive episode with a Hamilton Depression Rating Scale (HAMD) score > 17 and YMRS score < 7, and being medication-naïve or medication-free for at least 6 months; and (c) clinically remitted BD, defined as HAMD-17 scores < 7 and YMRS scores < 5 across 4 consecutive visits with no significant current manic or depressive symptoms. First-episode depression patients met criteria for MDD, had a HAMD-17 score ≥ 17 and YMRS score ≤ 5, with illness duration ≤ 2 years and no prior use of psychotropic medications. HC participants were recruited from the community and via public postings on the internet. HC participants were required to have no history of mental illness, family history of mental illness, or history of psychotropic substance use. This standardized inclusion framework ensured diagnostic rigor and cohort comparability across clinical groups (Fig. 2).

#### Exclusion criteria

Exclusion criteria were as follows: (a) meeting *DSM-5* diagnostic criteria for any psychiatric disorder other than the 3 target diagnoses (MDD, SZ, or BD), including but not limited to pervasive developmental disorders, intellectual disability, anxiety disorders, eating disorders, or other Axis I conditions; (b) meeting criteria for alcohol or drug dependence, or having a history of substance abuse (excluding caffeine and nicotine) within the past 3 months; (c) having severe physical illnesses such as hypertension, diabetes, cardiovascular or cerebrovascular disease, or any unstable systemic condition; (d) having a history of brain or cervical spine surgery, severe head trauma with loss of consciousness, epilepsy, febrile seizures, coma, or any organic brain lesion; (e) using antibiotics within the past 3 months; (f) receiving modified electroconvulsive therapy within the past 6 months; (g) being pregnant or breastfeeding; (h) having a history of gastrointestinal or other major digestive disorders; and (i) having any neurological or systemic condition known to affect cognitive function.

### Data collection

#### Clinical and neurocognitive evaluation

General information was collected by trained clinicians and included a wide range of human phenotypic characteristics, such as gender, age, education level, handedness, age of onset, disease duration, past medical history, dietary habits, sleep patterns, and family history. The following tools were employed:

HAMD-17 [25]: Widely used in clinical practice, this scale has good application reliability and is commonly used to assess the severity of depressive states in patients for the current time and the previous week. Total score less than 7: no depressive state; score 7 to 17: possible depressive state; score 18 to 24: depressive; score greater than 24: severe depressive.

Hamilton Anxiety Scale (HAMA) [26]: This scale consists of 14 items and is an important clinical assessment tool. HAMA can effectively reflect the severity of anxiety symptoms in patients for the current time and the previous week. Total score less than 7: no anxiety symptoms; score greater than or equal to 14: definite anxiety; score greater than or equal to 21: marked anxiety; score greater than or equal to 29: severe anxiety.

YMRS [27]: Primarily used to assess mania symptoms and severity. Result criteria: 0 to 5: normal; 6 to 12: mild mania; 13 to 19: moderate mania; 20 to 29: severe mania; over 30: extreme mania.

PANSS [28]: Composed of 7 positive symptoms, 7 negative symptoms, and 16 general psychopathological symptoms, totaling 30 items, with 3 additional items assessing the risk of aggression.

MATRICS Consensus Cognitive Battery (MCCB) [29]: Assesses processing speed, attention/vigilance, working memory, verbal learning and memory, visual learning and memory, reasoning and problem-solving, and social cognition.

The aforementioned scales are administered by trained and qualified psychiatrists who strictly follow the guidelines and scoring criteria. Assessments are based on all information from the week prior to evaluation, relying on live conversation, observation, and informant information.

#### Blood samples

Both patient and HC groups provide 5 ml of venous blood samples upon enrollment for testing. Within a 48-h window from clinical data collection, ensuring patients have fasted for over 8 h, venous blood is collected and sent to the Department of Laboratory Medicine at The Affiliated Brain Hospital of Guangzhou Medical University for testing. Remaining venous blood samples are tested for laboratory markers, with serum separation within 12 h, centrifugation at 3,000 rpm at room temperature for 10 min, separating the supernatant plasma and the pelleted blood cells, both of which are stored at −80 °C for future use.

#### EEG samples

Resting-state EEG recordings were acquired by trained technicians in a sound-attenuated, dimly lit room. Participants sat comfortably with eyes closed for 10 min and were instructed to remain relaxed, avoid excessive movement, and stay awake. Scalp activity was captured with a 32-channel Ag/AgCl Neuroscan system (international 10–20 montage) at a sampling rate of 1,000 Hz. Pz served as the reference, and an additional ground electrode was placed at AFz. Electrode impedances were maintained below 10 kΩ throughout acquisition, and continuous monitoring allowed real-time adjustment if impedance exceeded this threshold. Before recording, participants removed metallic accessories and were screened for recent caffeine or nicotine intake to minimize physiological artifacts. Raw EEG traces were visually inspected online to mark gross artifacts or drowsiness; these segments were subsequently excluded during preprocessing. All data were timestamped, anonymized, and stored in a secure research server for further analysis.

#### MRI scans

The MRI images of subjects in phase 1 and 2 were collected using a Siemens Prisma 3.0T MRI scanner, while those of subjects in Periods 3, 4, and 5 were collected using a Philips 3.0T MRI scanner. Both equipment are located at the Department of Radiology, The Affiliated Brain Hospital of Guangzhou Medical University. Scanning operations are performed by at least one professional radiologist and one professional psychiatrist. This study collects resting-state MRI image data from participants, who are instructed to keep their eyes closed but remain awake and relaxed, minimizing head and swallowing movements. Participants are provided with specialized nonmagnetic headphones and a head restraint to reduce interference and head movement. MRI scans include the following: (a) Resting-state fMRI scans. Gradient-echo planar imaging (GRE-EPI) sequence is used to acquire rs-fMRI images, with the following scan parameters: repetition time = 800 ms; echo time = 30 ms; flip angle = 56°; field of view = 208 × 208 mm^2^; voxel size = 2 × 2 × 2 mm^3^; multiband scanning parallel to the anterior–posterior commissure; a total of 72 slices and 450 time points collected per participant. (b) T1 scans. MPRAGE sequence is used to acquire T1 images, with the following scan parameters: repetition time = 2,000 ms; echo time = 2.32 ms; flip angle = 8°; field of view = 230 × 230 mm^2^; matrix = 256 × 256; slice thickness = 0.9 mm. After acquisition, all participants’ DICOM images are checked by 2 radiologists to ensure no missing data and obvious artifacts before preprocessing.

#### Fecal samples

Participants were instructed to fast for 12 h prior to sample collection and provided both fecal and venous blood samples between 7:00 and 9:00 AM the next morning. All samples were collected aseptically by trained research assistants. Fecal samples were collected using sterile containers and spoons, immediately sealed, and divided into 3 aliquots. To preserve sample integrity, they were transferred to a −80 °C freezer within 30 min. Dietary data were obtained using a 3-day 24-h recall and food frequency questionnaire, documenting the intake of major food groups and calculating nutrient intake. For metagenomic sequencing, microbial DNA was extracted through combined chemical and mechanical lysis, followed by phenol-chloroform purification, RNase treatment, and ethanol precipitation. After quality assessment, DNA was fragmented (~300 to 400 bp), and libraries were constructed via end repair, A-tailing, adaptor ligation, and PCR amplification. Whole-genome shotgun sequencing was performed on the Illumina platform to generate high-quality raw data for downstream analysis.

### Statistical analysis

Continuous variables were expressed as mean ± standard deviation (SD), while categorical variables were summarized as counts and percentages. The normality of continuous variables was assessed using the Shapiro–Wilk test, and homogeneity of variance was determined via the Levene test. Group comparisons for categorical variables were performed using the chi-square test. For continuous variables, independent-sample *t* tests were used when the data met assumptions of normality and homogeneity of variance; otherwise, the nonparametric Mann–Whitney *U* test was applied. For comparisons involving 3 or more groups, either one-way analysis of variance or the Kruskal–Wallis *H* test was employed based on data distribution, followed by Tukey’s or Dunn’s post hoc tests when necessary. When multiple comparisons were performed, *P* values were adjusted using the Benjamini–Hochberg method to control the false discovery rate. All statistical analyses were conducted using R or SPSS software, with *P* value < 0.05 considered statistically significant.

### Research progress

Over the course of 6 years, we established a dataset comprising 1,266 participants, including 893 patients with psychiatric disorders: 509 with MDD (of whom 230 were treatment-naive, first-episode patients), 63 with BD, and 321 with acute exacerbations of SZ. The mean disease duration among patients was 53 months. Additionally, 373 HC were recruited. Detailed demographic and clinical characteristics are summarized in Table 2. Additionally, we conducted structured assessments of participants’ lifestyle habits and health status at both baseline and follow-up stages. These assessments included information on medication adherence, dietary patterns, gastrointestinal symptoms, antibiotic use history, daily activity levels, and exercise frequency. The incorporation of these scales facilitates effective control for lifestyle confounders in subsequent analyses. The relevant follow-up questionnaires and scale contents are fully documented in the Supplementary Materials.

**Table 2.** Summary of BIGHI substudy characteristics. For detailed results of the intergroup comparisons, please refer to Table S6.

Characteristics	Group A (FEMDD)	Group B (MDD)	Group C (BD)	Group D (SZ)	Group E (HC)
Gender (M/F)	67/163	66/213	26/37	197/124	185/188
Age, years	23.07 ± 5.05	24.17 ± 11.83	35.08 ± 13.20	36.79 ± 14.29	29.14 ± 12.01
Years of education	14.34 ± 2.67	11.93 ± 3.32	12.63 ± 3.41	10.89 ± 3.36	14.42 ± 2.91
BMI	20.56 ± 3.52	20.88 ± 3.42	24.15 ± 5.43	–	22.25 ± 3.76
PANSS	62.29 ± 10.12	–	40.44 ± 9.73	72.05 ± 23.44	–
P	10.41 ± 3.02	–	8.59 ± 2.47	17.56 ± 8.02	–
N	14.76 ± 4.06	–	10.37 ± 4.69	19.99 ± 8.19	–
G	37.08 ± 5.78	–	21.49 ± 4.77	34.49 ± 10.93	–
HAMD	23.42 ± 4.89	26.57 ± 5.50	3.27 ± 5.08	–	–
HAMA	19.60 ± 5.80	23.39 ± 6.49	3.56 ± 7.14	–	–
YMRS	3.47 ± 4.06	4.46 ± 4.63	1.65 ± 3.33	–	–
BACS-SC	24.50 ± 9.50	–	41.89 ± 15.75	34.27 ± 13.353	44.23 ± 13.66
CFT	56.19 ± 9.97	–	43.33 ± 22.69	37.51 ± 20.40	57.10 ± 11.92
TMT	31.27 ± 10.99	–	40.97 ± 14.32	36.20 ± 15.14	44.77 ± 11.18
HVLT-R	32.57 ± 9.79	–	42.46 ± 14.99	32.67 ± 12.59	43.52 ± 9.46
BVMT-R	40.32 ± 8.47	–	45.41 ± 16.83	35.16 ± 12.28	46.89 ± 9.35
WMS	39.06 ± 11.09	–	43.36 ± 14.29	36.05 ± 13.58	47.73 ± 10.17
CPT	35.04 ± 9.77	–	44.56 ± 13.01	36.49 ± 10.84	46.04 ± 9.95

FEMDD, first-episode major depressive disorder; MDD, major depressive disorder; BD, bipolar disorder; SZ, schizophrenia; HC, healthy controls; BMI, body mass index; PANSS, Positive and Negative Syndrome Scale; HAMD, Hamilton Depression Rating Scale; HAMA, Hamilton Anxiety Scale; YMRS, Young Mania Rating Scale; BACS-SC, Brief Assessment of Cognition in Schizophrenia-Symbol Coding; CFT, Category Fluency Test-Animal Naming; TMT, Trail Making Test; HVLT-R, Hopkins Verbal Learning Test-Revised; BVMT-R, Brief Visuospatial Memory Test-Revised; WMS, Wechsler Memory Scale; CPT, Continuous Performance Test

### Future cohort expansion

BIGHI is grounded in real-world clinical cohorts and is designed to support the systematic expansion of sample size and accumulation of multimodal data within an established framework for cross-system and cross-diagnostic joint modeling. Core clinical assessments and symptom rating scales are uniformly collected across all participants, with EEG, MRI, gut microbiome profiles, and peripheral biomarkers forming the primary multimodal data layers. Longitudinal follow-up is conducted around key clinical time windows using a structured yet flexible design compatible with real-world clinical practice. Further details regarding sample size planning, multimodal overlap, and follow-up design are provided in the Supplementary Materials.

## Main Research Content

Dysregulation of the MGBA may constitute a cross-diagnostic mechanism underlying multiple categories of psychiatric disorders. Based on this core hypothesis, BIGHI conducted a series of exploratory studies during the cohort development phase, focusing on different methodologies and diagnostic groups. These studies spanned multiple dimensions including EEG, MRI, gut microbiome, and integrative multi-omics analysis, providing preliminary clues at different systemic levels regarding how MGBA abnormalities manifest biologically and correlate with clinical symptoms. Collectively, this exploratory work establishes a preliminary framework for understanding the multidimensional biological architecture of psychiatric disorders.

### EEG-based characterization and biomarker discovery in major psychiatric disorders

**Fig. 3.**
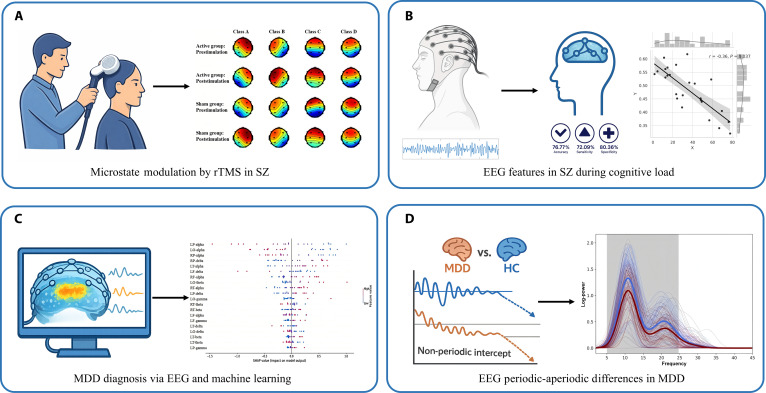
EEG-based investigations and biomarker insights in psychiatric disorder research. (A) Microstate modulation under rTMS reveals therapeutic effects. Through EEG microstate analysis, we observed significant changes in microstate duration in chronic SZ patients following rTMS treatment. (B) Nonlinear dynamics and network features derived from cognitive load EEG reveal frontal lobe dysfunction and support machine learning classification for SZ. By extracting multi-frequency nonlinear dynamics features and whole-brain network metrics from EEG under cognitive task load, frontal lobe dysfunction associated with cognitive deficits can be identified. These EEG features simultaneously provide robust support for machine learning classification. (C) Analysis of EEG spectral features captures abnormal cortical regulatory dynamics in MDD, with alterations in left parietal alpha-band power emerging as an important discriminative electrophysiological feature. (D) Analysis of periodic and aperiodic EEG features reveals marked differences between MDD and HC. By decomposing EEG signals into periodic and aperiodic components, we observed a characteristic reduction in the aperiodic component in MDD, which was also associated with symptom severity. Panels (A) and (D) are reproduced with permission from the original publishers, while panel (C) is reprinted from an open-access article under the terms of the Creative Commons Attribution License (CC BY 4.0).

From an electrophysiological perspective, EEG enables the characterization of dynamic neural activity patterns in individuals with psychiatric disorders, facilitating the identification of aberrant neural activity features and potential electrophysiological correlates. Through EEG microstate analysis, we evaluated the therapeutic effects of repetitive transcranial magnetic stimulation (rTMS) on positive symptoms in chronic SZ patients [30]. Over a 4-week treatment period, patients received active or sham 10 Hz stimulation targeting the left dorsolateral prefrontal cortex (DLPFC) for 5 sessions per week. Active rTMS resulted in a significant reduction in the duration of microstate C and a marked increase in microstate D, indicating that 10 Hz stimulation can ameliorate positive symptoms in chronic SZ and that EEG microstates may serve as sensitive indicators of symptom improvement (Fig. 3A). Furthermore, we calculated features such as nonlinear dynamics across different frequency bands of EEG and brain functional network properties, analyzed EEG signals of SZ patients under cognitive load, and revealed the association between frontal lobe functional impairment and cognitive deficits in SZ. We constructed an automatic classification method based on these EEG features, achieving an accuracy rate of 76.77%, a sensitivity of 72.09%, and a specificity of 80.36% in classifying SZ patients (Fig. 3B).

In studies of MDD, integrating EEG spectral features with machine learning has identified an abnormal pattern of cortical activity that is thought to indirectly reflect dysregulated cortical excitability. MDD patients showed markedly reduced relative α-band power across the parieto-occipital region, attenuated scalp functional connectivity in the α band, and enhanced γ-band connectivity. Notably, reduced α-band power in the left parietal region emerged as a key electrophysiological marker of MDD [31] (Fig. 3C). Recently, by comparing EEG data from patients with MDD and HC, we observed that in the MDD group, there was a significant reduction in alpha wave power in the parieto-occipital region and beta wave power in the right parieto-occipital area. Moreover, the nonperiodic intercept in the parieto-occipital region of the MDD group was significantly lower and positively correlated with the HAMD scores (Fig. 3D), suggesting that this nonperiodic component may serve as a potential biomarker for depression [32]. Additionally, we observed systematic changes in EEG microstate features across different severity levels of MDD symptoms. MDD patients generally exhibited reduced microstate A and increased microstate C, while those with severe MDD also demonstrated prolonged microstate D, suggesting compensatory overactivation of the executive control network. As symptoms worsened, microstate transition flexibility further declined, and microstate D emerged as a potential neural indicator of depression severity [33]. When comparing MDD patients with and without suicidal ideation (SI) to HC, a significant reduction in microstate D was observed, manifested as decreased coverage and fewer transitions from A to D. These alterations accompany slowed processing speed, suggesting that impaired cognitive control functions may represent a key neural mechanism underlying SI [34]. Collectively, these findings highlight EEG’s potential for identifying brain abnormalities and aiding psychiatric diagnosis.

### MRI-based mapping of brain networks and biomarkers in major psychiatric disorders

**Fig. 4.**
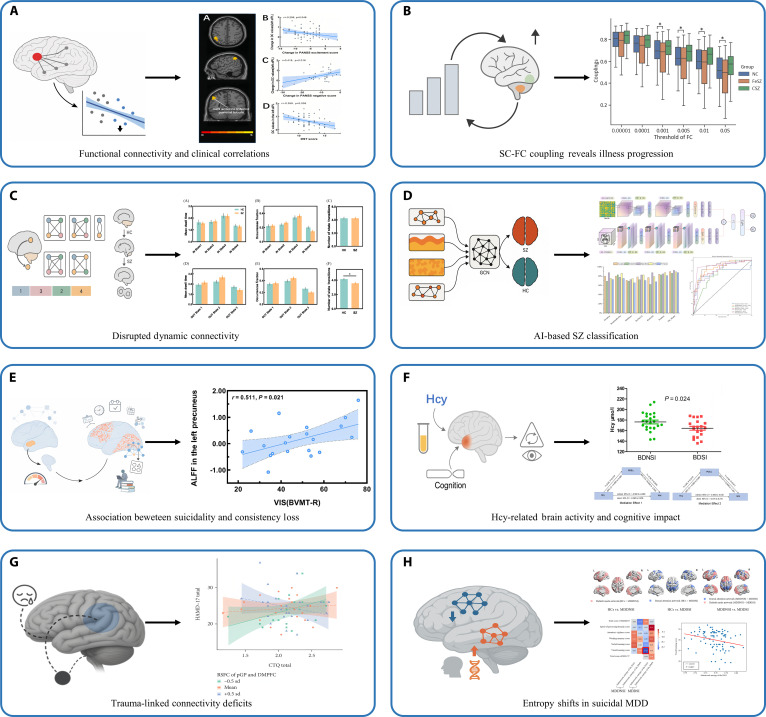
Overview of neuroimaging-based approaches a in psychiatric disorder research. (A) Functional connectivity and symptom associations. In SZ, alterations in functional connectivity of key brain regions, before and after treatment, correspond systematically to clinical symptom improvement, indicating that the plasticity of functional networks can serve as a marker of therapeutic effects. (B) Stage-specific abnormalities in structural–functional coupling. By integrating structural and functional connectivity measures, SZ patients at different illness stages exhibit distinct coupling-strength patterns. Structural–functional coupling not only differentiates disease stages but also relates to overall symptom burden. (C) Disrupted dynamic connectivity. SZ patients show a clear reduction in the transition frequency of dynamic effective connectivity between the cerebellar dentate nucleus and the cerebral cortex. This reduction reflects diminished flexibility in their neural communication and is closely related to cognitive impairments. (D) AI-based SZ classification. By integrating multiple MRI-derived metrics, including functional connectivity, amplitude of low-frequency fluctuations, regional homogeneity, and degree centrality into a graph convolutional network model, we achieved a robust classification of SZ and HC. (E) Consistency loss in BD with suicidal ideation. BD patients with suicidal ideation show markedly reduced voxel-wise consistency in key temporal, occipital, and somatosensory regions, and these alterations are closely associated with impairments in working memory. (F) Group differences in Hcy levels between BDSI and BDNSI, and their link to brain activity, suggest peripheral metabolic indicators may influence cognition by modulating brain regions. (G) Trauma-linked connectivity deficits in MDD. In adolescents with MDD, functional connectivity between the posterior putamen and the dorsomedial prefrontal cortex is markedly reduced. This decrease shows a systematic association with the extent of childhood adversity, where lower connectivity corresponds to more severe depressive symptoms. These findings suggest that early-life trauma may disrupt the development of frontostriatal circuits. (H) Entropy shifts in suicidal MDD. Patients with suicidal ideation show decreased entropy within the dorsal attention network and increased entropy within the default mode network, reflecting altered information complexity and network stability. These patterns are associated with cognitive function and immune-related gene expression. Panels (A), (B), (C), and (E) are reproduced with permission from the original publishers, while panels (D), (F), (G), and (H) are reprinted from open-access articles under the terms of the Creative Commons Attribution License (CC BY 4.0).

Through the integration of structural and functional MRI, we systematically mapped brain network alterations across diagnostic groups, aiming to pinpoint key regions and connectivity features linked to symptoms, cognition, and illness stage. In terms of SZ, we constructed brain functional networks in patients following antipsychotic treatment and found that changes in the degree centrality of the left anterior inferior parietal lobule were negatively correlated with changes in positive scores (Fig. 4A). Furthermore, the post-treatment degree centrality of this region was significantly negatively associated with performance on the Symbol Digit Modalities Test [35]. Subsequently, we constructed structural–functional brain connectivity maps for first-episode drug-naive SZ, chronic SZ, and HC, and calculated the structural–functional (SC–FC) coupling strength. The results showed that, at the global connectivity level, chronic SZ patients had significantly higher SC–FC coupling strength than first-episode patients. At the node-strength level, first-episode SZ patients exhibited markedly lower coupling strength than HC, and this reduction was positively correlated with PANSS negative symptom scores [36] (Fig. 4B). In addition, by calculating dynamic effective connectivity, we also found that the dynamic connectivity patterns between the cerebellar dentate nucleus and the cerebral cortex in SZ patients were significantly different from those in HC, exhibiting a marked reduction in transition frequency (Fig. 4C), which was associated with neurocognitive functions [37]. As part of our work on intelligent diagnosis, we computed key MRI-based features, including amplitude of low-frequency fluctuations (ALFF), regional homogeneity (ReHo), and degree centrality, and further analyzed large-scale functional connectivity patterns. Using these multimodal features, we constructed a GCN model to classify SZ and HC. Compared with traditional machine learning and deep learning approaches that rely solely on regional MRI features, integrating functional MRI with connectomics markedly improved classification performance. The model achieved an accuracy of 92.47%, with areas under the receiver operating curve (AUC), sensitivity, specificity, precision, and F1 score of 95.36%, 88.70%, 95.06%, 92.87%, and 90.45%, respectively [38]. Additionally, we classified SZ patients and HC using 3 brain atlases and 5 classifiers, validating the classification performance with 2 cross-validation methods. The results indicated that the whole-brain atlas with 268 ROIs outperformed the other 2 brain atlases [39]. We also classified SZ patients and HC using an SVM model based on gray matter volume (GMV) and white matter features, achieving an accuracy of 88.4%. Notably, reductions in GMV and alterations in white matter among SZ patients were primarily observed in regions implicated in emotion, memory, and visual processing [40]. Furthermore, we developed an integrated deep learning model that combines 3D and 2D convolutional neural network (CNN) architectures to classify SZ patients using multimodal MRI features. In this framework, 3D images of T1, ReHo, and ALFF were used as inputs to the 3D CNN, whereas SC and FC matrices were used as inputs to the 2D CNN. SE blocks were incorporated, and the softmax layer was replaced with an SVM classifier. The resulting integrated model achieved an accuracy of 89.86%, demonstrating strong potential for improving automated SZ diagnosis [41] (Fig. 4D).

We also conducted studies on BD and MDD. Using resting-state functional connectivity analysis, we found that patients with BD who exhibited suicidal ideation showed significantly reduced voxel-wise consistency in the left fusiform gyrus, left lingual gyrus, right middle temporal gyrus, and left postcentral gyrus compared to those without suicidal ideation. Additionally, in patients with suicidal ideation (Fig. 4E), voxel-wise consistency in the left middle occipital gyrus was negatively correlated with working memory performance [42]. In addition, we examined the relationship among plasma homocysteine (Hcy) levels, brain function, and cognition in BD patients with suicidal ideation (BDSI) and without suicidal ideation (BDNSI). Hcy levels were significantly lower in the BDSI group and positively correlated with fALFF signals in the left posterior cingulate gyrus (Fig. 4F), which further mediated the negative association between Hcy and visual/verbal learning performance [43]. In adolescent MDD patients, we observed a significant reduction in FC between the left posterior putamen and the right dorsomedial prefrontal cortex compared with HC. Further moderation analysis showed that this aberrant FC modulated the relationship between childhood maltreatment and the severity of adolescent-onset MDD symptoms. Interaction analysis additionally revealed that when FC was lower, childhood maltreatment exhibited a stronger positive association with MDD symptom severity [44] (Fig. 4G). In addition, we constructed an edge-centric functional network and compared node entropy between MDD patients with and without suicidal ideation. Patients with suicidal ideation showed reduced subnet entropy in the dorsal attention network and increased entropy in the default mode network. Node-entropy features effectively distinguished the 2 groups, and the observed alterations were associated with the expression of genes involved in cell signaling and immune-inflammatory processes (Fig. 4H). These alterations were associated with visual learning performance and the expression of genes related to immune and inflammatory responses [45].

### Dysbiosis of the gut microbiome in major psychiatric disorders

**Fig. 5.**
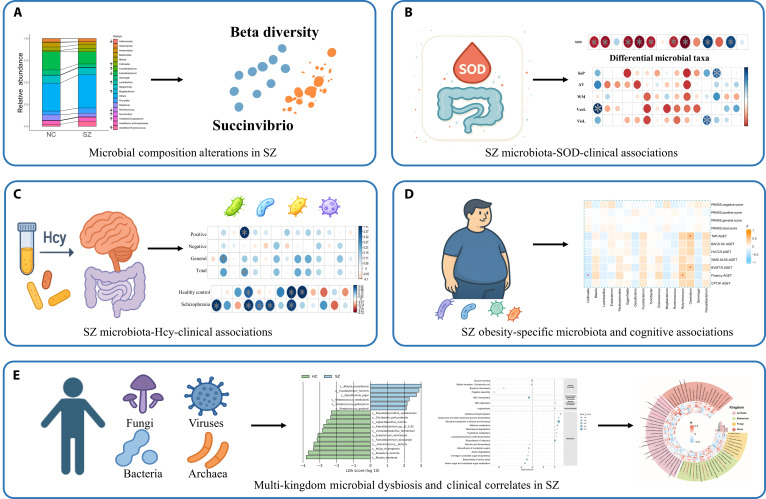
Overview of gut microbiota alterations and their clinical associations. (A) Compared with HC, SZ patients show significant differences in gut microbial community structure, beta diversity, and genus-level abundance. The abundance of *Succinivibrio* is significantly associated with clinical symptom measures. (B) SOD levels in SZ patients are significantly associated with the abundance of several differential gut microbes, including *Collinsella* and *Faecalibacterium*, as well as with performance in verbal learning. In addition, the abundance of *Faecalibacterium* and related taxa shows a positive association with visual learning and other cognitive function scores. (C) Hcy levels in SZ patients are markedly altered and correlate with the abundance of multiple gut microbial groups, including *Lactobacillus*, *Lactobacillaceae*, and *Bifidobacterium*. The abundance of *Lactobacillus* is positively associated with disease severity. (D) SZ patients with comorbid obesity exhibit distinct patterns of gut microbial composition. Elevated *Collinsella* abundance is negatively associated with cognitive test performance, whereas reduced abundance of *Clostridium* and *Butyricicoccus* is positively associated with several cognitive function scores. (E) SZ patients show significant alterations in the composition and metabolic functional profiles of microbial communities across bacteria, archaea, fungi, and viruses. The figure presents abundance differences of these microbial taxa and their correlations with symptom ratings and cognitive function. Panels (B), (C), and (D) are reproduced with permission from the original publishers, while panels (A) and (E) are reprinted from open-access articles under the terms of the Creative Commons Attribution License (CC BY 4.0).

At the microbiological level, we comprehensively evaluated gut microbiota alterations in major psychiatric disorders to uncover structured associations among microbial profiles, metabolic processes, and clinical symptoms. We found significant alterations in the gut microbiota of patients with SZ [46], including alterations in β-diversity, increases in specific taxa such as *Collinsella* and *Lactobacillus*, and disruptions in multiple metabolic pathways such as pantothenate and CoA biosynthesis. Among these microbial features, the abundance of *Succinivibrio* was positively associated with symptom severity, whereas *Corynebacterium* showed a negative association (Fig. 5A). We also collected cognitive function, blood biochemical indicators, and gut microbiome data from patients with SZ, focusing on the relationships between oxidative stress markers such as superoxide dismutase (SOD) and Hcy with disease risk and cognitive decline. Machine learning models were further applied to evaluate the potential of SOD as a biomarker. Our findings showed that peripheral SOD levels were generally elevated in SZ patients and accompanied by pronounced gut microbial dysbiosis. The abundances of *Collinsella*, *Desulfovibrio*, and other taxa were increased, whereas beneficial genera such as *Faecalibacterium*, *Anaerostipes*, *Turicibacter*, and *Ruminococcus* were markedly reduced. SOD levels were systematically associated with the abundances of *Eubacterium*, *Collinsella*, and *Lactobacillus*, as well as with cognitive performance, suggesting that oxidative stress may influence brain function through microbiome-related pathways (Fig. 5B) [47]. Another study further demonstrated that Hcy levels were significantly higher in SZ patients compared with HC and were closely associated with poorer verbal learning performance. In addition, Hcy levels showed positive correlations with several gut microbial taxa, including *Lactobacillus*, *Bifidobacterium*, *Corynebacterium*, *Mogibacterium*, and *Bulleidia* (Fig. 5C) [48]. In an obesity-specific study of SZ, significant alterations in gut microbiota composition and metabolic profiles were observed. Notably, increased abundance of *Collinsella* was significantly negatively associated with cognitive function (Fig. 5D), while decreased levels of *Clostridium* and *Butyricicoccus* were positively associated with cognitive function [49]. Moreover, studies focusing on nonbacterial gut microbes remain scarce. Metagenomic analysis revealed pronounced dysbiosis across multiple microbial kingdoms in SZ patients, encompassing bacteria, fungi, archaea, and viruses, and identified 21 significantly altered metabolic pathways that showed strong species-function coordination. In addition, the abundance of *Streptococcus vestibularis* was positively correlated with Hcy levels (Fig. 5E) [50].

### Gut–brain associations in major psychiatric disorders

**Fig. 6.**
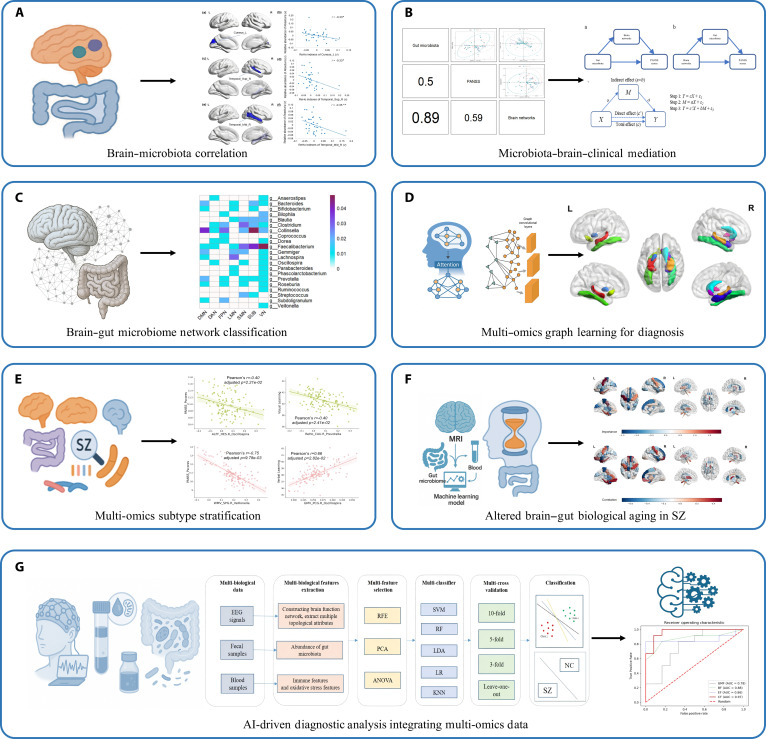
Integrated brain–gut microbiome analyses reveal clinical relevance and predictive biomarkers. (A) Correlation analysis between brain functional indices and gut microbial abundance shows multiple significant associations in SZ. Regional homogeneity values in areas such as the right superior temporal sulcus and the left precuneus exhibit negative correlations with the abundance of *Ruminococcaceae*, with a distributed pattern across regions. (B) Mediation modeling using gut microbial abundance as the independent variable and brain network node properties as mediators identifies several regions involved in visual, language, and motor processing whose node characteristics show statistically significant associations between microbial abundance (e.g., *Selinomonas*) and symptom rating scores. (C) A brain–gut network constructed by integrating neuroimaging features with gut microbial profiles effectively distinguishes SZ from HC. Feature importance mapping indicates that *Faecalibacterium* is associated with functional indices in visual and subcortical regions, while *Collinsella* corresponds to indicators within the default mode and subcortical networks. (D) MO-GCN integrating brain imaging and gut microbiome features, combined with an attention mechanism for feature weighting, achieves improved classification performance and identifies key feature groups associated with symptom severity and cognitive function. (E) Multi-omics subtype analysis identifies distinct subtypes across brain, gut, and integrated brain–gut feature spaces. Subtypes derived from brain and brain–gut features show clearer correspondence to symptom profiles, whereas gut-based subtypes align more closely with cognitive test outcomes. (F) A biological-age prediction model incorporating MRI, gut microbiome, and blood-based features demonstrates a significantly increased biological age in SZ relative to chronological age. The figure presents the relationships between biological age gap, cognitive scores, and symptom severity. (G) An AI-driven diagnostic framework integrating EEG, blood biochemical metrics, and gut microbiome features achieves high-accuracy SZ classification, with the best performance obtained through multi-feature fusion. Panels (A) to (G) are reprinted from open-access articles distributed under the terms of the Creative Commons Attribution License (CC BY 4.0).

To explore cross-system mechanisms of the MGBA, we further integrated multimodal datasets to identify how interactions across systems jointly shape symptomatology and cognitive function. In extending this framework to SZ, we examined the multidimensional associations between the gut microbiome and brain imaging features, uncovering potential interactions underlying disease manifestations (Fig. 6A). At the genus level, we observed markedly reduced abundances of *Ruminococcus* and *Roseburia* and an increased abundance of *Veillonella* in SZ patients. Consistently, MRI analyses revealed decreased GMV and ReHo, together with increased ALFF across multiple brain regions. Gut microbial α-diversity was significantly linked to both measures, and ReHo in the right superior temporal cortex, left cuneus, and right middle temporal gyrus was negatively associated with the abundance of *Roseburia* [51]. To further explore the connection between the MGBA and psychiatric symptoms, we performed mediation analysis using gut microbiome as the independent variable. Node properties from 7 brain regions, mainly in visual, language, and motor systems (Fig. 6B), significantly mediated the association between *Selinomonas* abundance and PANSS scores in SZ patients [52]. In terms of data integration, we established, for the first time, an individualized brain–gut–microbiome network (BGMN) by combining multimodal MRI features with gut microbiome profiles. This model achieved high accuracy in distinguishing SZ patients from HC. Among the most discriminative features, *Faecalibacterium* was associated with MRI-derived features in visual and subcortical regions, while *Collinsella* correlated with the default mode and subcortical networks (Fig. 6C). Additionally, working memory performance was positively associated with the connectivity between the right inferior parietal lobule and *Collinsella*, suggesting the potential of BGMN for biomarker discovery in SZ [53]. Leveraging brain–gut network methodology, we further developed a multi-omics graph convolutional network (MO-GCN) and incorporated an attention mechanism to more effectively integrate multi-omics features. The best classification accuracy reached 84.0%. Interpretability analysis showed that the most informative brain features were primarily related to memory and emotional regulation, such as those in the hippocampus and olfactory cortex, while the key microbial features were mainly from *Dorea* and *Ruminococcus*. These important features were significantly associated with clinical disease severity and cognitive function [54] (Fig. 6D). Moreover, to address the marked heterogeneity of SZ, we employed a data-driven approach to identify distinct multi-omics subtypes across brain, gut, and brain–gut integrated features. The results revealed that brain and brain–gut subtypes were more strongly associated with symptom profiles, while gut-derived subtypes exhibited closer links to cognitive function (Fig. 6E), highlighting the multilayered heterogeneity of SZ and offering potential pathways for precision diagnosis and treatment [55]. Building upon the multi-omics phenotyping that reveals the multi-layered heterogeneity of SZ, we further extend our analytical framework to the biological age dimension. Recently, we constructed a biological age prediction model using MRI, gut microbiome, and blood-based data. Unlike traditional single-modality biological age estimates, the multimodal approach substantially improved prediction accuracy. Patients with SZ showed a markedly increased biological age gap, which was closely associated with cognitive impairment and disease severity (Fig. 6F), highlighting its potential value in psychiatric assessment [56]. Finally, by integrating gut microbiome profiles, blood-based biomarkers, and EEG network features into a unified multi-omics framework, we substantially improved the diagnostic performance for SZ. The optimized model achieved an accuracy of 91.7% and an AUC of 96.5%. Notably, the most informative features spanned multiple biological domains, including microbial genera (e.g., *Lactobacillus* and *Prevotella*), peripheral biomarkers (SOD and immune cell ratios), and EEG-derived measures of nodal efficiency and local network integration within temporal and frontoparietal regions [57] (Fig. 6G). In studies on MDD, recent research has uncovered a critical coupling mechanism between cognitive impairment in first-episode MDD and gut microbiome dysbiosis. Notably, *Amycolatopsis* sp. Hca4 exhibits significant correlations with cognitive abilities such as processing speed. It influences working memory by modulating functional connectivity between the medial frontal gyrus and parahippocampal gyrus, suggesting that specific microorganisms may serve as pivotal nodes regulating abnormal brain networks and cognitive impairment [58]. These findings highlight the key role of gut–brain interactions in understanding, diagnosing, and treating major psychiatric disorders.

## Discussion

To the best of our knowledge, BIGHI is the first prospective cohort in China dedicated to investigating the MGBA in psychiatric disorders. The BIGHI cohort has several notable strengths. First, it encompasses a wide diagnostic spectrum by systematically enrolling individuals with MDD, SZ, BD, and HC. Second, we implemented standardized protocols with stringent quality control to collect comprehensive data, including clinical assessments, neurocognitive measures, dietary records, EEG, MRI, blood biomarkers, and fecal metagenomic sequencing profiles, along with longitudinal follow-up. This design not only fills a critical gap in domestic research but also overcomes key limitations of previous international studies, such as relatively limited sample sizes, single-diagnosis focus, and cross-sectional designs.

Our study underscores that psychiatric disorders are highly complex and heterogeneous conditions, with distinct pathological features emerging across different omics layers, such as the gut microbiome, neuroimaging, EEG signals, and blood biomarkers. These preliminary findings underscore the systemic nature of psychiatric disorders and emphasize the limitations of single-modality approaches in adequately explaining the complexity of these conditions [59]. The integration of multidimensional data offers new perspectives and pathways for the early identification, subtype classification, and precision intervention of psychiatric disorders [60,61]. In addition, the analysis strategy rigorously controlled key clinical confounders, including age, sex, body mass index, dietary patterns, medication exposure, and lifestyle factors, in order to minimize the influence of nondisease factors on biomarker characteristics [15,62]. Furthermore, we explored the integration of AI, with an emphasis on machine learning and deep learning methods, into psychiatric research. By integrating AI techniques, we can investigate potential biomarkers within the MGBA, such as alterations in functional connectivity and specific gut microbial signatures, to identify early-stage psychiatric conditions and facilitate timely subtyping and precision treatment [63,64]. As the cohort expands to multiple centers, independent external datasets will be used for validation to systematically evaluate model generalizability and ensure its transferable clinical applicability.

Against this backdrop, the BIGHI initiative demonstrates significant scientific value. Leveraging real-world data from individuals with multiple psychiatric disorders and a multimodal data system, this cohort is evolving into China’s first psychiatric disorders research platform capable of both broad-spectrum diagnosis and longitudinal follow-up [65]. More importantly, changes across all levels of the MGBA can be mapped to clinical symptom dimensions, enabling us to reinterpret the pathological structure of psychiatric disorders from a systems biology perspective rather than being confined to single-modality or single-system explanatory frameworks [66,67]. To address persistent challenges in severe psychiatric disorders, including unclear mechanisms, ambiguous subtypes, and unpredictable treatment responses, the multimodal design of BIGHI enables progress in 3 major directions: first, integrating gut microbiota, MRI, EEG, blood markers, and clinical features to decipher cross-system interaction patterns and their structural associations with symptom dimensions [59]; second, systematically comparing shared mechanisms and disease-specific pathways across psychiatric disorders to identify core MGBA pathways with cross-diagnostic significance and support the theoretical framework of the mental disorder continuum [68,69]; and third, leveraging longitudinal follow-up to map MGBA dynamic remodeling patterns during disease onset, progression, and intervention response, thereby identifying key biomarkers predictive of disease course and treatment response [70].

From a mechanistic perspective, existing research on the mechanisms of psychiatric disorders increasingly recognizes the bidirectional regulatory properties of the MGBA, which may influence brain function through neural, immune, and metabolic pathways [9,11]. At the microbiome level, potentially harmful bacteria associated with inflammatory responses and impaired gut barrier function (e.g., *Collinsella*) show an upward trend, whereas short-chain fatty acid-producing genera such as *Faecalibacterium* and *Roseburia* consistently decrease across different diagnostic groups [49,69]. This dysbiotic pattern parallels elevated peripheral inflammation, disrupted tryptophan metabolism, and altered lipid metabolic profiles, suggesting potential pathogenic mechanisms [71,72]. Neuroimaging studies similarly reveal convergent abnormalities in functional connectivity across multiple psychiatric disorders, characterized by strengthened positive coupling between the anterior cingulate cortex and the insula, along with weakened negative connectivity with the posterior parietal cortex and the lateral occipital cortex [73]. However, cross-modal integration of neuroimaging and microbiome data remains notably heterogeneous, and stable, reproducible mechanistic interpretations are still lacking. In addition, combining microbiome data with blood-based inflammatory, metabolic, and neurotransmitter-related biomarkers can enhance the biological interpretability of associations and provide important clues for inferring potential causal pathways within the MGBA [47,48]. Nevertheless, deeper mechanistic validation remains constrained by clinical ethical limitations.

In parallel, ongoing advances in animal experiments and synthetic biology [74–76] have further expanded our understanding of the MGBA mechanism and provided new directions for its therapeutic application in psychiatric disorder. It has been found that transplantation of fecal microbiota from patients with SZ or depression into germ-free mice induces behavioral phenotypes similar to those of psychiatric disorders, such as social withdrawal, cognitive deficits, and emotional abnormalities, suggesting that the MGBA may harbor an underlying causal mechanism [77,78]. However, the process involves complex biological pathways at multiple levels and still needs to be elucidated by systematic studies. It is worth emphasizing that early intervention is of great clinical value in the treatment of psychiatric disorders [79]. Animal experiments have shown that administration of psychobiotics to mice modeling chronic stress significantly alleviated brain dysfunction and anxiety-depression-like behaviors, suggesting a positive intervention potential [80,81]. On this basis, synthetic biology-driven engineered bacteria strategy is also emerging, which is expected to achieve more precise regulation of MGBA by modifying intestinal strains to synthesize neuromodulators such as GABA and 5-HT [82]. Meanwhile, traditional Chinese medicine prescriptions, such as the “xiaoyaosan” has shown potential in regulating the homeostasis of the intestinal flora, which provides a multifaceted therapeutic strategy that integrates the traditional and modern approaches to psychiatric disorders [83]. In the future, how to construct an effective mapping between human MGBA and animal models [84,85], and how to carry out cross-species and mechanism-oriented research will be the key path to promote MGBA from basic discovery to clinical translation [86].

This study has several limitations. First, BIGHI is currently in a single-center development phase with relatively concentrated sample sources, which may affect the external generalizability of results. Future efforts will gradually expand to multi-center collaboration to enhance geographic and population representativeness. Second, influenced by clinical visit patterns and disease spectrum distribution, sample sizes remain imbalanced across diagnostic categories, limiting statistical power for cross-disease comparisons. Additionally, low follow-up compliance among psychiatric patients means some longitudinal data are still being accumulated, preventing a more systematic analysis of disease progression dynamics in the current study. Although multimodal clinical and biological data have been collected, the cohort has not yet incorporated host genomic information, making it impossible to systematically evaluate the interaction mechanisms between genetic risk and environmental factors. Future integration of whole-genome sequencing and multi-omics data will facilitate the construction of a more comprehensive “host genome–microbiome–brain network–symptoms” mechanistic model. As the cohort expands across multiple centers and follow-up data matures, it will increasingly realize its potential for mechanistic elucidation and clinical translation in MGBA research on psychiatric disorders.

## Data Availability

The access of the dataset presented in this paper is available on request to the corresponding authors.
